# PDZD8-deficient mice manifest behavioral abnormalities related to emotion, cognition, and adaptation due to dyslipidemia in the brain

**DOI:** 10.1186/s13041-023-01002-4

**Published:** 2023-01-19

**Authors:** Yuji Kurihara, Kotone Mitsunari, Nagi Mukae, Hirotaka Shoji, Tsuyoshi Miyakawa, Michiko Shirane

**Affiliations:** 1 Department of Molecular Biology, Graduate School of Pharmaceutical Sciences, Nagoya City University, Nagoya, Aichi Japan; 2 Division of Systems Medical Science, Center for Medical Science, Fujita Health University, Toyoake, Aichi Japan

**Keywords:** PDZD8, Knockout mouse, Behavior, Dyslipidemia, Lipophagy, Cholesterol

## Abstract

Although dyslipidemia in the brain has been implicated in neurodegenerative disorders, the molecular mechanisms underlying its pathogenesis have been largely unclear. PDZD8 is a lipid transfer protein and mice deficient in PDZD8 (PDZD8-KO mice) manifest abnormal accumulation of cholesteryl esters (CEs) in the brain due to impaired lipophagy, the degradation system of lipid droplets. Here we show the detailed mechanism of PDZD8-dependent lipophagy. PDZD8 transports cholesterol to lipid droplets (LDs), and eventually promotes fusion of LDs and lysosomes. In addition, PDZD8-KO mice exhibit growth retardation, hyperactivity, reduced anxiety and fear, increased sensorimotor gating, and impaired cued fear conditioned memory and working memory. These results indicate that abnormal CE accumulation in the brain caused by PDZD8 deficiency affects emotion, cognition and adaptive behavior, and that PDZD8 plays an important role in the maintenance of brain function through lipid metabolism.

**Supplementary Information:**

The online version contains supplementary material available at 10.1186/s13041-023-01002-4.

## Introduction

Dyslipidemia is associated with neurodegenerative disorders such as Alzheimer’s disease, Huntington’s disease, and Parkinson’s disease [1–4]. Intracellular cholesteryl esters (CEs) are either supplied by low density lipoprotein (LDL) or synthesized in the endoplasmic reticulum (ER) and stored in lipid droplets (LDs), which are degraded as needed and their metabolic products are reused. Excessive accumulation of lipids in the brain cause the generation of reactive oxygen species and lysosomal damage, leading to brain dysfunction. The mechanism by which CE accumulates in the brains of patients with neurodegenerative diseases had been unknown. However, it has recently been appeared that PDZD8 promotes constant degradation of CEs in the brain by lipophagy [5]. However, the detailed mechanism of the lipophagy, which depends on lipid transport activity of PDZD8, had not been fully understood.

PDZD8 is a component of membrane contact sites between ER and endolysosomes, where it forms a tethering complex with protrudin, VAP-A/B, and Rab7 [6–10]. Membrane contact sites are close appositions between two organelles which allow them the exchange of molecular information each other. They therefore play an important role in interorganelle communication, with their main specific functions including lipid transfer, calcium regulation, and control of organelle dynamics [11–13].

PDZD8 contains a synaptotagmin-like mitochondrial lipid binding protein (SMP) domain, which is a mammalian ortholog of the yeast ER mitochondrial encounter structure (ERMES) complex subunit Mmm1, and possesses lipid transfer activity [10, 14–16]. A key intracellular function of the PDZD8 complex is transporting lipids between ER and Rab7-positive organelles such as endolysosomes and thereby promoting endosomal maturation and maintaining neuronal integrity [10, 17–23]. PDZD8 also functions in the regulation of ER-mitochondrial interactions and calcium dynamics in neurons [24].

Mutations of the PDZD8 gene in humans have been associated with intellectual disability (ID) [25], providing further support for an important role of PDZD8 in the brain. Intellectual disability is a type of neurodevelopmental disorder that affects 2% to 3% of the general population and is characterized by marked impairment of cognitive ability and adaptive behavior [26–29]. The phenotype of *Pdzd8*^tm1b^ mice with a mutation similar to those identified in humans with *PDZD8*-related ID were recently reported [25]. These mice harbor a deletion of exon 3 of *Pdzd8*, which is located downstream of the coding sequence for the SMP domain. A PDZD8 protein fragment including the transmembrane (TM) and SMP domains may therefore be expressed in these *Pdzd8*^tm1b^ mice. The behavioral phenotype of *Pdzd8*^tm1b^ mice is characterized by spontaneous stereotypies, decreased anxiety, increased exploration, and impaired spatial memory.

In contrast, we have generated mice completely deficient in gene expression of PDZD8 including SMP domain with the use of the CRISPR-Cas9 system. We had previously shown that PDZD8 promotes lipophagy via SMP domain-dependent cholesterol transport and that CEs abnormally accumulate in the brain of PDZD8-deficient (PDZD8-KO) mice due to impaired lipophagy [5]. However, the detailed mechanism of lipophagy via cholesterol transfer by PDZD8 had not been fully elucidated. The behavioral phenotype of PDZD8-KO mice had not been also unknown.

Here we demonstrate that PDZD8 promotes cholesterol transfer to LDs, resulting in fusion of LDs with lysosomes, thereby promoting lipophagy. We also show that PDZD8-KO mice with abnormal accumulation of CEs in the brain exhibit abnormalities in emotion, cognition and adaptive behavior including restricted growth, hyperactivity, and decreased anxiety and fear, similar to *Pdzd8*^tm1b^ mice [25]. In addition, we found that PDZD8-KO mice exhibit increased sensorimotor gating and reduced cued fear conditioned memory and working memory, which are novel findings for phenotype of PDZD8-deficient mice. These results thus suggest that PDZD8 plays an important role in the maintenance of brain function through lipid metabolism.

## Results

### PDZD8 promotes fusion of LDs with lysosomes mediated by cholesterol

**Fig. 1:**
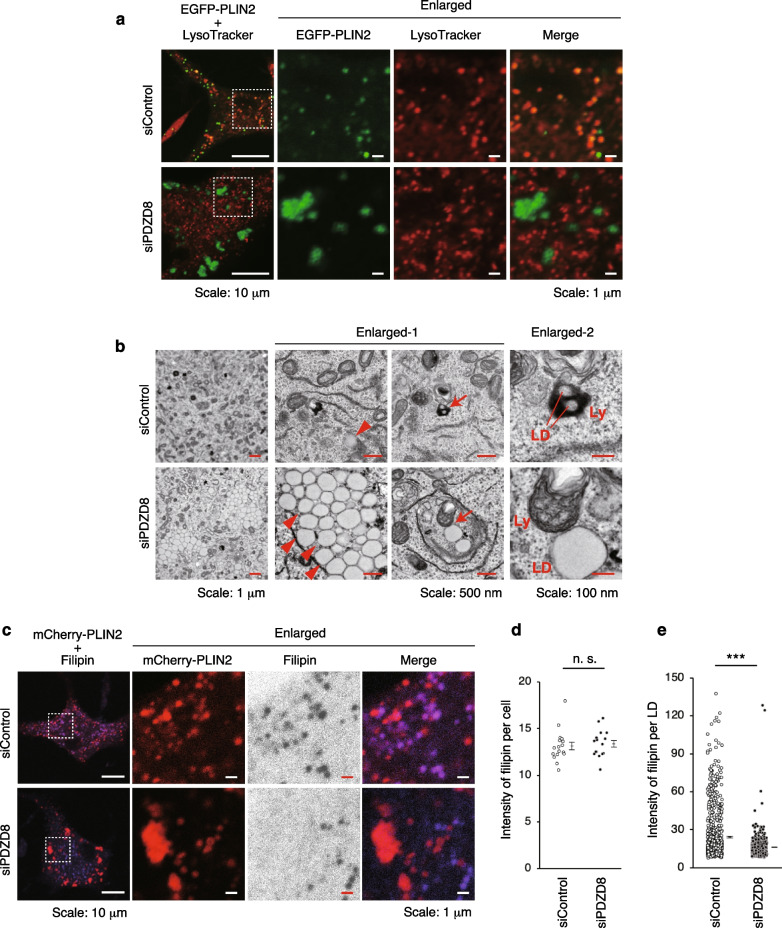
PDZD8 promotes fusion of LDs with lysosomes mediated by cholesterol. **a** Confocal fluorescence images of PC12 cells transfected with siControl or siPDZD8 as well as with expression vectors for EGFP-PLIN2 (green) and then metabolically labeled with LysoTracker (red). The boxed regions in the left panels are shown at higher magnification in those to the right. **b** TEM images of PC12 cells transfected with siControl or siPDZD8. Arrowheads in the images at higher magnification (enlarged-1) indicate LDs. Arrows in the enlarged-1 indicate organelles during lipophagy, which are shown at much higher magnification in those to the right (enlarged-2). LD and lysosome (Ly) are also indicated in the enlarged-2. **c** Confocal fluorescence images of PC12 cells transfected with siControl or siPDZD8 as well as with expression vectors for mCherry-PLIN2 (red) and then metabolically labeled with filipin (blue). The boxed regions in the left panels are shown at higher magnification in those to the right. Filipin is shown in gray scale in the enlarged images. **d** and **e** Intensity of filipin per cell (n = 17 and 16 cells for siControl and siPDZD8, respectively) (**d**) or per LD (n = 772 and 719 LDs for siControl and siPDZD8, respectively) (**e**) in images similar to those in (**c**). Quantitative data are presented with SEM values. n.s., not significant; ***P < 0.001

PDZD8-KO mice exhibit abnormal accumulation of CEs in the brain due to impaired lipophagy, which is progressed by the fusion of LDs and lysosomes [5]. However, the mechanism underlying the selective fusion of LDs with lysosomes had not been fully understood. We then examined the mechanism for the fusion of LDs and lysosomes during lipophagy in rat pheochromoctyoma PC12 cells. PC12 cells were transfected with control siRNA (siControl) or rat PDZD8 siRNA (siPDZD8), as well as an expression vector for the LD marker EGFP-PLIN2, and then labeled with lysosomal marker LysoTracker Red. As a result, siPDZD8-transfected cells specifically showed significant aggregation of LDs and the marked reduction of overlap between EGFP-PLIN2 and LysoTracker, as shown previously [5] (Fig. 1a). We then observed the detailed structure of LDs and lysosomes by TEM and found that single LDs were prominent in siControl cells, whereas aggregated LDs were prominent in siPDZD8 cells (Fig. 1b, arrowheads). Focusing on the fusion of LDs with lysosomes during lipophagy, LDs surrounded by lysosomes and under degradation were prominent in siControl cells, whereas intact LDs without fusion with lysosomes were prominent in siPDZD8 cells (Fig. 1b, arrow). From these results, the process of the fusion of LD and lysosomes is impaired during lipophagy in siPDZD8 cells.

We next investigated the mechanism on selective fusion of LDs with lysosomes during PDZD8-mediated lipophagy. Given that PDZD8, which binds to Rab7 via CC domain, transports cholesterol from ER to the Rab7-positive organelles [5], cholesterol facilitates membrane fusion of Rab7-positive organelles and autophagosomes [30], and Rab7 promotes lipophagy [31], we examined whether the fusion of LDs and lysosomes by PDZD8 depends on cholesterol. PC12 cells were transfected with siControl or siPDZD8, as well as an expression vector for the LD marker mCherry-PLIN2, and then labeled with cholesterol marker filipin. The overlap between mCherry-PLIN2 and filipin was remarkable in siControl cells, whereas the overlap was barely detectable in siPDZD8 cells (Fig. 1c). Statistical analysis of these results showed that there was no difference in the fluorescence intensity of filipin per cell between siControl and siPDZD8 cells (Fig. 1d), but the intensity of filipin at LD was significantly lower in siPDZD8 cells than siControl cells (Fig. 1e). Thus, the distribution of cholesterol to LDs was significantly reduced in siPDZD8 cells. These results suggest that PDZD8 promotes the fusion of LDs and lysosomes by cholesterol transfer to LDs, thereby promoting lipophagy.

### Generation of PDZD8-KO mice

**Fig. 2:**
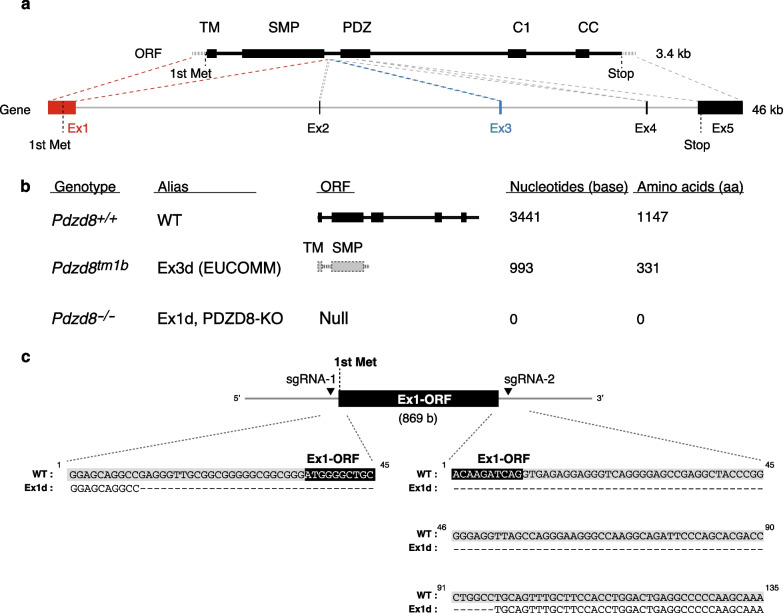
Gene targeting of the mouse *Pdzd8* locus. **a** Schematic representation of the domain structure of PDZD8, the corresponding ORF, and the genomic structure of the *Pdzd8* locus. Exons (Ex) and introns of the gene are shown as boxes and lines, respectively. **b** Schematic representation of the domain structure for the ORF products of wild-type (WT), Ex3d (from EUCOMM) [25], and Ex1d alleles of *Pdzd8*. The genotype, genotype abbreviation, as well as corresponding numbers of nucleotides and amino acids are also shown. **c** Schematic representation of the genomic structure around exon 1 of *Pdzd8* showing the positions targeted by the two sgRNAs for generation of the Ex1d allele. The sequences of the WT and Ex1d alleles are also shown, with exon 1 and the adjacent upstream and intron sequences being highlighted in black and gray, respectively

For generation of PDZD8-deficient mice with a null mutation, we designed 5' and 3' single guide RNAs (sgRNAs) targeted to the open reading frame (ORF) of exon 1 of the PDZD8 gene, which contains the start codon and encodes the TM domain and most of the SMP domain, and deleted it with the CRISPR-Cas9 system (Fig. 2a). This exon 1 deletion of PDZD8 gene (Ex1d) become null mutation because there is no start codon (Additional file 1: Fig. S1a). In contrast, in the embryonic stem cells from EUCOMM from which *Pdzd8*^tm1b^ mice were derived, the PDZD8 gene was deleted by insertion of loxP cassettes flanking each side of exon 3. Given that exon 3 is located downstream of the coding sequence for the SMP domain, a PDZD8 protein fragment initiated at the start codon and including the TM and SMP domains could be produced from the mutant locus (Fig. 2a, Additional file 1: Fig. S1a). The ORF remaining after this exon-3 deletion (Ex3d) has a stop codon 1 base after the deleted sequence and therefore comprises a total of 993 bases (equivalent to 331 amino acids) (Additional file 1: Fig. S1b). Thus, whereas Ex1d is a null mutation, Ex3d may be not (Fig. 2b). The sequence of the Ex1d allele was confirmed to completely lack the ORF of exon 1 including the initiating methionine between the sites targeted by the two sgRNAs (Fig. 2c).

### PDZD8-KO mice show growth retardation

Given that PDZD8 is a risk factor for syndromic ID with autistic features [25] and for PTSD [32] in humans, we performed a battery of behavioral tests to examine the possible effects of PDZD8 ablation on physiological endophenotypes with the use of 18 wild-type (WT) and 19 PDZD8-KO male mice on the C57BL/6 J background that were obtained as a single cohort by heterozygote intercrossing.

**Fig. 3:**
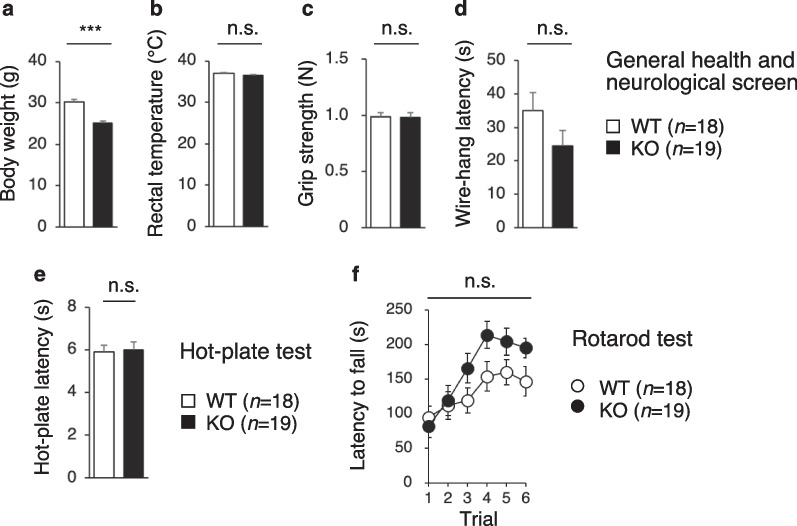
General health and neurological screen of PDZD8-KO mice. **a**, **b** Body weight (**a**) and body temperature (**b**) of male WT and PDZD8-KO mice at 10 to 20 weeks of age. **c** Grip strength. **d** Latency to fall in the wire-hang test. **e** Latency of the first paw response in the hot-plate test of pain sensitivity. **f** Latency to fall from the rotating rod in the rotarod test of motor function. All data are means ± s.e.m. (WT mice, *n* = 18; PDZD8-KO mice, *n* = 19). *P* values for differences between genotypes were determined with Student’s *t* test (**a**, **c**), the Mann–Whitney U test (**b**, **d**, **e**), or two-way repeated-measures ANOVA (**f**). ***P < 0.001; n.s., not significant. The results of all statistical analysis are provided in Additional file 2: Table S2

We first examined the general health of, and performed a neurological screen for, these mice at 10 to 20 weeks of age. PDZD8-KO mice showed a reduced body weight that was ~ 83% of that of WT mice (Fig. 3a), suggestive of restricted growth, but they had a normal body temperature (Fig. 3b). Muscular strength as assessed with the grip strength test and the wire-hang test did not differ significantly between WT and PDZD8-KO mice (Fig. 3c, d). Analysis of pain sensitivity with the hot-plate test revealed no abnormality in PDZD8-KO mice (Fig. 3e). Assessment of motor coordination and motor learning with the rotarod test, in which the time for mice to fall from a rotating cylinder is measured, also showed no abnormality in PDZD8-KO mice (Fig. 3f). Together, these results thus revealed growth retardation but no major abnormalities in general or neurological health in PDZD8-KO mice.

### PDZD8-KO mice manifest reduced anxiety and fear

**Fig. 4:**
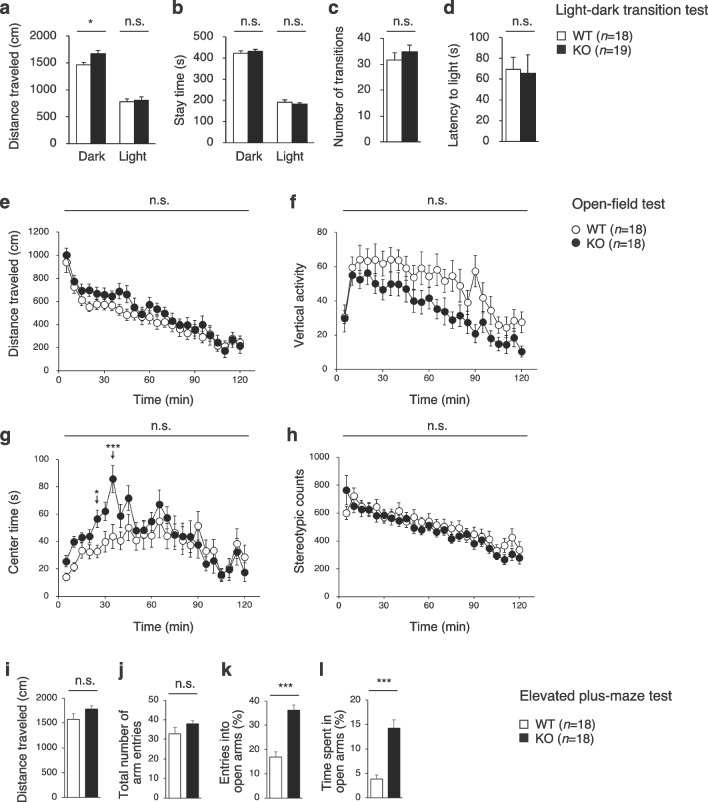
Anxiety and fear-related behavior of PDZD8-KO mice. **a–d** Distance traveled in the light and dark chambers (**a**), time spent in the light and dark chambers (**b**), number of transitions between the light and dark chambers (**c**), and latency of entry into the light chamber (**d**) for the light–dark transition test. **e**–**h**, Distance traveled (**e**), vertical activity (**f**), center time (**g**), and stereotypic counts (**h**) for the open-field test. **i**–**l**, Distance traveled (**i**), total number of arm entries (**j**), percentage of entries into the open arms (**k**), and percent time spent in the open arms (**l**) for the elevated plus-maze test. All data are means ± s.e.m. (WT mice, *n* = 18; PDZD8-KO mice, *n* = 19 for **a**–**d** and *n* = 18 for **e**–**l**). *P* values for differences between genotypes were determined with Student’s *t* test (**a**–**c**, **k**), the Mann–Whitney U test (**d**, **i**, **j**, **l**), or two-way repeated-measures ANOVA (**e**–**h**). *P < 0.05, ***P < 0.001; n.s., not significant. The results of all statistical analysis are provided in Additional file 2: Table S2

To evaluate anxiety-related behavior, we performed the light–dark transition test, the open-field test, and the elevated plus-maze test. The light–dark transition test, which is based on the premise that mice have an aversion to a brightly illuminated area, revealed no significant difference in the distance traveled in the light chamber, time spent in the light chamber, number of transitions between the light and dark chambers, or latency to enter the light chamber between WT and PDZD8-KO mice (Fig. 4a–d). However, the distance traveled in the dark chamber was greater for PDZD8-KO mice than for WT mice, suggestive of hyperactivity in the mutant animals.

In the open-field test, which is based on the premise that mice have an aversion to open and illuminated spaces, we found that distance traveled, vertical activity, time spent in the center area, and stereotypic counts did not differ significantly between WT and PDZD8-KO mice over the entire examination period (Fig. 4e–h). However, time spent in the center area during the intervals from 20 to 25 min and from 30 to 35 min was greater for PDZD8-KO mice than for WT mice, suggestive of a reduced level of anxiety in the former animals. The results of both the light–dark transition test and the open-field test also suggested that PDZD8-KO mice are normal with regard to locomotor activity.

The elevated plus-maze test assesses anxiety in an open space and fear of height on the basis of the movement of a mouse in an elevated apparatus consisting of four arms, two with walls and two without. This test did not reveal a significant difference in distance traveled (Fig. 4i) or the total number of arm entries (Fig. 4j) between WT and PDZD8-KO mice. However, the percentage of entries into the open arms (Fig. 4k) and percent time spent in the open arms (Fig. 4l) were significantly higher for PDZD8-KO mice than for WT animals, suggestive of reduced anxiety- and fear-related behaviors in the mutant animals.

### PDZD8-KO mice have defective fear conditioned memory

**Fig. 5:**
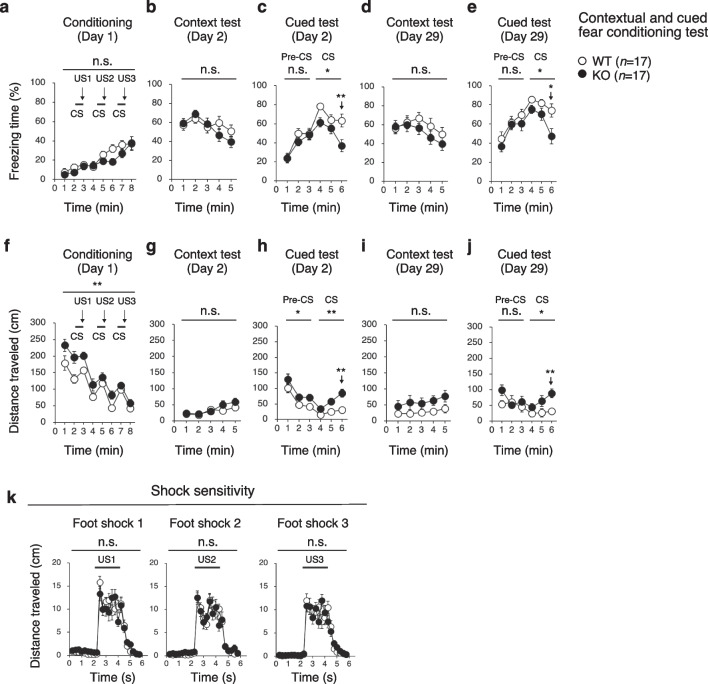
Contextual and cued fear memory of PDZD8-KO mice. **a**–**j**, Percent freezing time (**a**–**e**) and distance traveled (**f**–**j**) during conditioning (**a**, **f**), in a context test performed 1 day (**b**, **g**) or 28 days (**d**, **i**) after conditioning, and in a cued test with altered context performed 1 day (**c**, **h**) or 28 days (**e**, **j**) after conditioning in the contextual and cued fear conditioning test. Mice were presented three times with white noise as a conditioned stimulus (CS) for 30 s (horizontal black bars) followed by foot shock as an unconditioned stimulus (US) for the last 2 s of the conditioned stimulus (vertical arrows) during the conditioning session. **k** Shock sensitivity assessed on the basis of the distance traveled during and after exposure to the unconditioned stimulus. All data are means ± s.e.m. (*n* = 17 for WT and PDZD8-KO mice). Statistical analysis of differences between genotypes was performed with two-way repeated-measures ANOVA (**a**–**k**). For the cued test (**c**, **e**, **h**, **j**), separate *P* values are shown for the first and second 3-min periods as well as for the time point of 6 min (vertical arrows). *P < 0.05, **P < 0.01; n.s., not significant. The results of all statistical analysis are provided in Additional file 2: Table S2

The contextual and cued fear conditioning test assesses fear-related learning and memory. For conditioning (day 1), each mouse was placed in a chamber and presented three times with a conditioned stimulus of white noise followed by an unconditioned stimulus of mild foot shock (Fig. 5a, f). The distance traveled during conditioning was greater for PDZD8-KO mice than for WT mice, suggestive of hyperactivity in the mutant animals again (Fig. 5f). A contextual test and subsequent cued test were then performed. Freezing time and distance traveled were monitored to evaluate contextual memory and cued memory after both a short interval (1 day) and a long interval (28 days) relative to conditioning (Fig. 5b–e, g–j). Reactivity to the unconditioned stimulus appeared normal in PDZD8-KO mice (Fig. 5k). Although there was no significant difference in freezing time or distance traveled in the context test between the two genotypes (Fig. 5b, d, g, i), PDZD8-KO mice manifested a significantly reduced freezing time (Fig. 5c, e) and increased distance traveled (Fig. 5h, j) in the cued test compared with WT mice. Though PDZD8-KO mice showed no significant abnormalities for the entire 5 min in the contextual fear conditioning test by the Two-way repeated measures ANOVA, they represented reduced fear at several individual time points (Additional file 2: Table S2). These results suggested that PDZD8-KO mice are almost normal with regard to contextual memory but have a significant deficits in cued fear conditioned memory.

### PDZD8-KO mice show enhanced sensorimotor gating

**Fig. 6:**
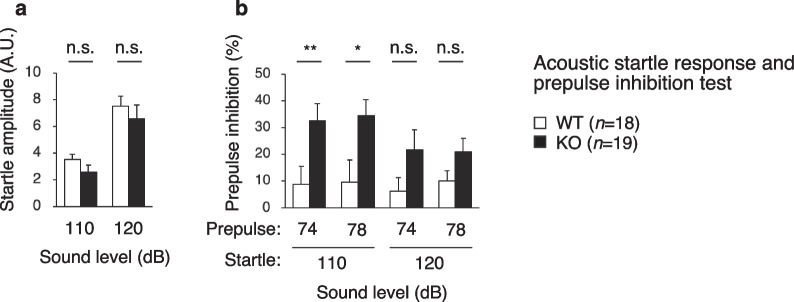
Acoustic startle response and prepulse inhibition test. Startle amplitude (A.U., arbitrary units) (**a**) and prepulse inhibition (**b**) were determined in the acoustic startle response and prepulse inhibition test. All data are means + s.e.m. (WT mice, *n* = 18; PDZD8-KO mice, *n* = 19). Statistical analysis of differences between genotypes was performed with Student’s *t* test (**a**, for 120 dB; **b**, for 78/110, 74/120, and 78/120 dB) or the Mann–Whitney U test (**a**, for 110 dB; **b**, for 74/110 dB). *P < 0.05, **P < 0.01; n.s., not significant. The results of all statistical analysis are provided in Additional file 2: Table S2

The acoustic startle response and prepulse inhibition test assesses sensorimotor gating and provides a measure of attention. Prepulse inhibition refers to the suppression of a startle response to a sudden strong sensory stimulus by a preceding weak sensory stimulus, and provides an indicator of defective information processing associated with schizophrenia. The startle response to a single loud sound in PDZD8-KO mice was normal (Fig. 6a). In contrast, prepulse inhibition was significantly increased in PDZD8-KO mice (Fig. 6b), suggestive of enhanced sensorimotor gating or increased habituation in the mutant animals.

### PDZD8-KO mice are hyperactive

**Fig. 7:**
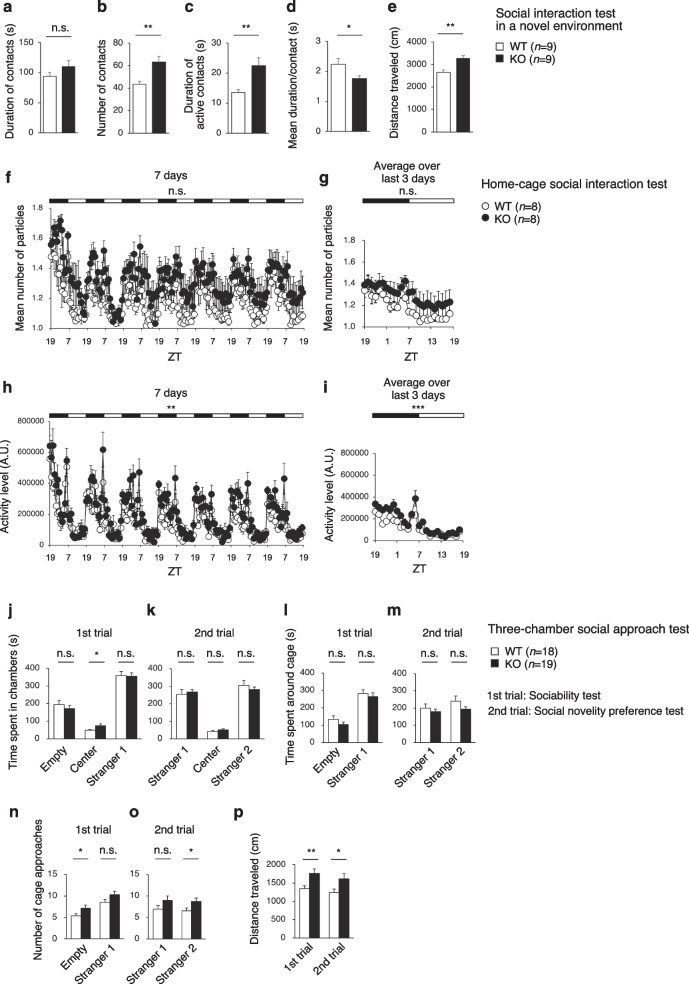
Social behavior of PDZD8-deficient mice. **a**–**e** Duration of contacts (**a**), number of contacts (**b**), duration of active contacts (**c**), mean duration per contact (**d**), and distance traveled (**e**) in the social interaction test of social behavior in a novel environment. Data are means + s.e.m. (*n* = 9 for WT and PDZD8-KO mice). **f**–**i**, Mean number of particles calculated for each hour over 7 days (**f**) and averaged over the last 3 days (**g**) as well as mean activity level (A.U., arbitrary units) for each hour over 7 days (**h**) and averaged over the last 3 days (**i**) in the home-cage social interaction test of social behavior in a familiar environment. Time is indicated in Zeitgeber time (ZT). Data are means ± s.e.m. (*n* = 8 for WT and PDZD8-KO mice). **j**–**p**, The time spent in the chamber containing an empty cage, in the center chamber, and in the chamber with a cage containing an unfamiliar mouse (stranger 1) (**j**); the time spent around each cage (**l**); the number of approaches to each cage (**n**); and the distance traveled (**p**) were determined in the three-chamber sociability test (1st trial). The time spent in the chamber containing caged stranger 1, the center chamber, and the chamber with a cage now containing a novel unfamiliar mouse (stranger 2) (**k**); the time spent around each cage (**m**); the number of approaches to each cage (**o**); and the distance traveled (**p**) were also determined in the three-chamber social novelty preference test (2nd trial). Data are means + s.e.m. (WT mice, *n* = 18; PDZD8-KO mice, *n* = 19). *P* values for differences between genotypes were determined with Student’s *t* test (**a**, **b**, **d**, **e**, **j**, **l**–**p**), the Mann–Whitney U test (**c**, **j**–**l**, **o**), two-way repeated-measures ANOVA (**f**–**i**), or Welch’s *t* test (**k**, **m**).*P < 0.05, **P < 0.01, ***P < 0.001; n.s., not significant. The results of all statistical analysis are provided in Additional file 2: Table S2

In the social interaction test, which assesses social behavior in a novel environment, two mice are placed in a cage and their behavior is monitored over a short time (10 min). The total duration of contacts in this test did not differ significantly between WT and PDZD8-KO mice (Fig. 7a), whereas the total number of contacts (Fig. 7b), duration of active contacts (Fig. 7c), and distance traveled (Fig. 7e) were significantly increased as well as mean duration per contact (Fig. 7d) were significantly decreased for PDZD8-KO mice. These results suggested that social interaction might be enhanced in PDZD8-KO mice, but they may also have been due to hyperactivity of the mutant animals.

In the home-cage social interaction test, which assesses social behavior in a familiar environment, two mice are placed in a cage and monitored for 7 days for determination of their activity level and mean number of contacts. Mice of the two genotypes did not show a significant difference in the mean number of particles (two particles indicate that the mice are not in contact with each other, whereas one particle indicates contact between the two mice) per hour either over 7 days (Fig. 7f) or averaged over the last 3 days (Fig. 7g). However, PDZD8-KO mice showed an increase in mean activity both over 7 days (Fig. 7h) and averaged over the last 3 days (Fig. 7i) compared with WT mice.

In the three-chamber social-approach test, a test mouse is placed in the center compartment of a three-chambered box containing an empty cage or a cage including an unfamiliar mouse (stranger 1) in the end chambers and its location is then monitored (sociability test, 1st trial). Another unfamiliar mouse (stranger 2) is then placed in the empty cage and the preference of the test mouse for the new stranger mouse is examined (social novelty preference test, 2nd trial). PDZD8-KO mice did not differ significantly from WT mice with regard to the time spent in the chamber containing stranger 1 or in the time spent around, or the number of times they approached, the cage containing stranger 1 in the 1st trial, but they did approach the cage containing stranger 2 in the 2nd trial more often than did WT mice (Fig. 7j–o). On the other hand, the distance traveled in each trial was significantly longer for PDZD8-KO mice than for WT mice (Fig. 7p). Overall, the results of these various types of social-behavior test suggested that PDZD8-KO mice are hyperactive rather than more sociable.

### PDZD8-KO mice do not manifest depression-like behavior

**Fig. 8:**
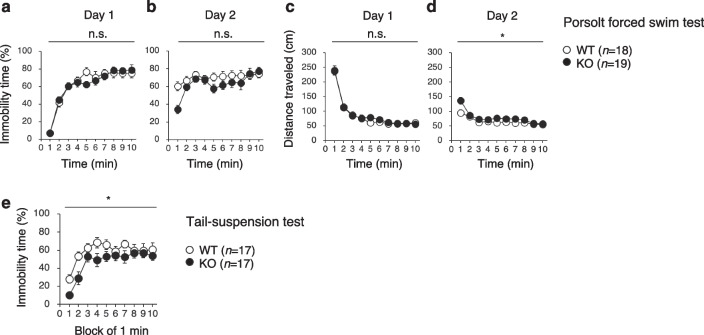
Absence of depression-related behavior in PDZD8-deficient mice. **a**–**d** Percentage immobility time (**a**, **b**) and distance traveled (**c**, **d**) on day 1 (**a**, **c**) and day 2 (**b**, **d**) in the Porsolt forced swim test. **e** Percentage immobility time in the tail-suspension test. All data are means ± s.e.m. (WT mice, *n* = 18 in **a**–**d** and *n* = 17 in **e**; PDZD8-KO mice, *n* = 19 in **a**–**d** and *n* = 17 in (**e**). *P* values for differences between genotypes were determined by two-way repeated-measures ANOVA. *P < 0.05; n.s., not significant. The results of all statistical analysis are provided in Additional file 2: Table S2

In the Porsolt forced swim test performed on two consecutive days, immobility time on day 1 (Fig. 8a) and day 2 (Fig. 8b) as well as distance traveled on day 1 (Fig. 8c) did not differ significantly between WT and PDZD8-KO mice, whereas distance traveled on day 2 (Fig. 8d) was greater for PDZD8-KO mice. These results suggested that PDZD8-KO mice do not exhibit depression-like behavior but are hyperactive. PDZD8-KO mice showed no significant abnormalities for the entire 10 min in immobility time of the Porsolt forced swim test in the Two-way repeated measures ANOVA, but appeared hyperactive at several individual time points (Additional file 2: Table S2). In the tail-suspension test, PDZD8-KO mice showed a decreased immobility time compared with WT mice (Fig. 8e), again suggesting that the mutant animals are unlikely to have a depression-like impairment but are hyperactive.

### PDZD8-KO mice have impaired working memory

**Fig. 9:**
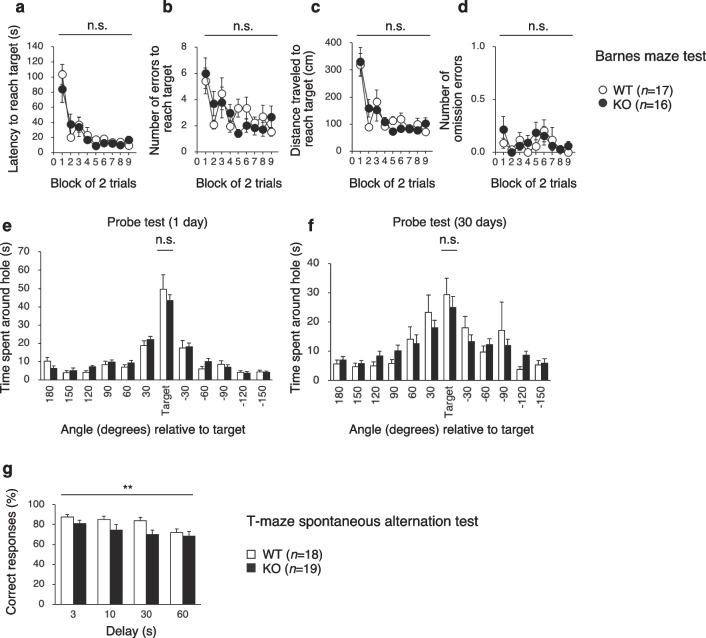
Learning and memory of PDZD8-KO mice. **a**–**d** Latency to reach the target hole (**a**) as well as the number of errors (**b**), distance traveled (**c**), and number of omission errors (**d**) before reaching the target hole during training sessions in the Barnes maze test of spatial-learning memory and remote memory. **e**, **f** Time spent around each hole in probe trials conducted 1 day (**e**) and 30 days (**f**) after the last training session of the Barnes maze test. All data for **a**–**f** are means ± s.e.m. (WT mice, *n* = 17; PDZD8-KO mice, *n* = 16). **g** Percent correct responses in the T-maze test of working memory. Data are means + s.e.m. (WT mice, *n* = 18; PDZD8-KO mice, *n* = 19). *P* values for differences between genotypes were determined by two-way repeated-measures ANOVA (**a**–**d**, **g**) or the Mann–Whitney U test (**e**, **f**). **P < 0.01; n.s., not significant. The results of all statistical analysis are provided in Additional file 2: Table S2

The Barnes maze test assesses spatial-learning memory and remote memory. Mice are trained to find a target box located under one of the holes in a maze for nine consecutive days (two trials per day, 18 trials in total). Neither the latency to reach the target hole (Fig. 9a) nor the number of errors (Fig. 9b), the distance traveled (Fig. 9c), or the number of omission errors (Fig. 9d) before reaching the target hole differed between WT and PDZD8-KO mice. To evaluate spatial-reference memory, we conducted probe trials without the escape box at 1 and 30 days after the last training trial. PDZD8-KO mice showed normal memory performance in both probe trials (Fig. 9e, f). These results indicated that PDZD8-KO mice are normal with regard to spatial-learning, spatial-reference, and remote memory.

Finally, to assess working memory, we performed the T-maze spontaneous alternation test. The percentage of correct responses was decreased for PDZD8-KO mice compared with WT mice (Fig. 9g), indicative of impaired working memory in the mutant animals.

## Discussion

We have here revealed that PDZD8 promotes cholesterol transfer to LDs, resulting in fusion of LDs with lysosomes during lipophagy. We also show that PDZD8-KO mice with dyslipidemia in the brain exhibit restricted growth, hyperactivity, decreased anxiety and fear, increased sensorimotor gating, and reduced cued fear conditioned memory and working memory. These results thus suggest that PDZD8 plays a role in the maintenance of brain function through lipid metabolism.

PDZD8-KO mice have abnormal CE accumulation in the brain but not in the liver, which reason may be that PDZD8 is highly expressed in the brain, or that lipid transport proteins other than PDZD8 may predominate in other tissues such as liver. In this study using the neuronal cell line PC12, more overlap of LD and lysosomes was observed in siControl cells, whereas less in siPDZD8 cells. In contrast, in a non-neuronal HeLa cells, there was no difference between siControl and siPDZD8 cells since even siControl cells show little overlap between LDs and lysosomes (data not shown). In addition, no LD aggregation was observed in siPDZD8 transfected HeLa cells so that the distribution of cholesterol to LD was not different between siControl and siPDZD8 in HeLa cells (data not shown). From these results, neuronal cells may need the constant degradation of LDs by lipophagy much more than non-neuronal cells. Since abnormal accumulation of lipids leads to an increase in reactive oxygen species and lysosome damage, impaired lipophagy in the brain may lead to brain dysfunction. Therefore, the behavioral abnormalities in PDZD8-KO mice shown in this study may be caused by lipid abnormalities in the brain.

PDZD8-KO mice exhibited a number of behavioral abnormalities related to emotion, cognition, and adaptation, suggesting that PDZD8 plays important roles in multiple regions of the brain. However, the exact reason why PDZD8 KO mice showed different degrees of abnormalities in the similar tests, such as contextual and cued fear-conditioned memory, is unknown. One of the possible explanation for these differences is that some neural circuits may be more affected by PDZD8, others less so. Actually, we examined the distribution of PDZD8 expression in the mouse brain in a previous paper and found that PDZD8 is highly expressed in many brain regions, such as striatum, medial habenula, amygdala, ventral tegmental area/substantia nigra pars reticulata, and trigeminal mesencephalic nucleus[5]. Abnormalities such as neurodegeneration in these brain regions of PDZD8-KO mice need to be investigated in detail in the future.

Pdzd8^tm1b^ mice (exon 3-deleted) show abnormalities such as restricted growth, spontaneous stereotypies, decreased anxiety, increased exploration, and impaired spatial memory [25]. In contrast, PDZD8-KO mice (exon 1-deleted) showed novel phenotypes such as increased sensorimotor gating and reduced cued fear conditioned memory and working memory, as well as the similar abnormalities to Pdzd8tm1b mice such as restricted growth, hyperactivity, and decreased anxiety and fear (Additional file 2: Table S1). One of the possible reason for these differences in behavior between two mouse lines is that much more behavioral analyses were performed for PDZD8-KO than for Pdzd8^tm1b^ mice. Another possible reason is that the SMP domain, which has lipid transport activity, is absent in PDZD8-KO but might be expressed in Pdzd8tm1b mice. However, there is no direct evidence for that Pdzd8^tm1b^ mice really express a truncated form of PDZD8 lacking the SMP domain. Such truncated proteins are often unstable and degraded as a result of structural abnormality, resulting in low expression of the proteins.

Behavioral correlates of anxiety and fear have been observed in many animal species, reflecting their importance as adaptations to potentially dangerous environments [33]. PDZD8-KO mice showed reduced anxiety and fear, but also reduced working memory, so it is unclear which of them is responsible for the reduced cued fear conditioned memory. Fear-conditioned memory is a defensive response triggered by anticipation of danger, and it is abnormal in individuals with posttraumatic stress disorder (PTSD) [34]. Working memory, on the other hand, refers to the temporary retention and manipulation of information during performance of cognitive tasks such as comprehension, learning, and reasoning, and it is impaired in children with neurodevelopmental disabilities [35]. Mutations of the PDZD8 gene in humans have been associated with intellectual disability (ID) due to neurodevelopmental disabilities [25], providing further evidence for an important role of PDZD8 in the brain. In addition, mutations in the PDZD8 gene in humans are reported to be a risk factor for PTSD [32]. While anxiety and fear are increased in PTSD, however, they are decreased in PDZD8-KO mice, which discrepancy cannot be explained at this time.

In conclusion, PDZD8 acts to promote lipophagy through cholesterol transport, and CEs abnormally accumulate in the brain of PDZD8-KO mice, resulting in behavioral abnormalities related to emotion, cognition, and adaptation in the mutant mice. PDZD8 is thus suggested to be an important protein for the maintenance of brain function.

## Methods

### Plasmids

Vectors encoding EGFP- and mCherrry-PLIN2 were described previously [5]. In brief, human cDNAs encoding PLIN2 was amplified from HeLa cells by PCR with specific primers and were then subcloned into pEGFP or pmCherrry (Clontech).

### Reagents

LysoTracker Red was from Thermo Fisher Scientific. Filipin complex from Streptomyces filipinensis was from Sigma-Aldrich.

### Cell culture and transfection

PC12 cells were cultured under a humidified atmosphere of 5% CO_2_ at 37 °C in Dulbecco’s modified Eagle’s medium (DMEM, Wako) supplemented with 10% fetal bovine serum (FBS, Nichirei) and on plates coated with poly-L-lysine (150–300 kDa, Sigma) before exposure to mouse submaxillary gland nerve growth factor (Merck Millipore) at 100 ng/ml in RPMI 1640 supplemented with 1% horse serum (Thermo Fisher Scientific). They were transfected with siRNAs or expression vectors with the use of a Nucleofector system (Lonza).

### RNA interference

Stealth siRNAs targeted to rat PDZD8 mRNA were obtained from Invitrogen–Life Technologies. The sequences of the siRNAs were 5'-GGGCCGGCTTAAAGTTACATTGCTA-3' (#1), 5'-CAGTCCCAAACGTACTCCAACAACA-3' (#2), and 5'-GAGGTGGCTTTAGGATGCCTAGCTA-3' (#3). The siRNAs were introduced into PC12 cells with the use of a Nucleofector instrument (Lonza), and the cells were then cultured for 72 h before experiments. As for both human and rat siRNAs, results are shown for siRNA #1, but similar findings were obtained with the other two siRNAs.

### Fluorescence imaging of live cells

Cells that had been transfected with plasmids encoding fluorescently tagged proteins or metabolically labeled with fluorescent probes were observed with an LSM800 confocal microscope (Zeiss), and the images were processed for calculation of fluorescence intensity with ZEN imaging software (Zeiss).

### TEM

Mouse brain was fixed with 2% glutaraldehyde (Nisshin EM) in 0.05 M cacodylate buffer containing 5 mM CaCl_2_. The fixed tissue was washed in cacodylate buffer with CaCl_2_, exposed to buffered 1% OsO_4_ plus 0.8% K_4_[Fe(CN)_6_]•3H_2_O for 1 to 2 h, dehydrated with acetone or ethanol, and embedded in Epon-Araldite or Spurr’s medium (Nisshin EM). Thin sections were stained with uranyl acetate and lead citrate and observed with a JEM-1400 Plus instrument (JEOL).

### Mutant mice

Generation of PDZD8-deficient mice was described previously [10]. In brief, Cas9 nickase mRNA (IDT 1074181, Alt-R S.p. Cas9 Nuclease 3NLS; IDT, San Jose, CA), Generic tracrRNA (IDT 1072532, Alt-R CRISPR-Cas9 tracrRNA), and target-specific crRNA (IDT, Custom Alt-R CRISPR crRNA) were mixed and injected into C57BL/6 J mouse zygotes with the use of a Super Electroporator NEPA21 Type II (Nepagene, Tokyo, Japan). The 5' and 3' single guide RNAs with 20-nucleotide sequences targeted to exon 1 (which contains the start codon) of the PDZD8 gene (GenBank accession number NM_001033222) and containing protospacer-adjacent motif sequences were 5'-GCAGGCCGAGGGTTGCGGCGGGG-3' and 5'-GCAGATTCCCAGCACGACCCTGG-3', respectively. Potential predicted off-target sites were checked for at http://crispr.mit.edu. The resultant mutant mice were backcrossed with C57BL/6 J mice before experiments. Animals were genotyped by PCR with the primers 5'-CTCAACAGACCCAGGAGAGG-3', 5'-AGCCCGACTTATCCAGGTCT-3', and 5'-CGCTGGAGACCTGCTACTTC-3' and with MightyAmp DNA polymerase (Takara, Shiga, Japan). All mouse experiments were approved by the animal ethics committee of Nagoya City University.

### General protocol for behavioral tests

WT and PDZD8-KO mice were group-housed (three or four animals per cage) in a room with a 12-h-light, 12-h-dark cycle (lights on at 7 a.m. and off at 7 p.m.) and with access to food and water ad libitum. Behavioral tests were performed between 9 a.m. and 6 p.m. with male mice at 10 to 20 weeks of age as described previously [36–40], unless indicated otherwise. Each apparatus was cleaned with hypochlorite solution before testing of each animal in order to prevent bias due to olfactory cues.

### Neurological screen

Mice were subjected to physical assessment, including measurement of rectal temperature and body weight. A wire-hang test apparatus (O’Hara & Co., Tokyo, Japan) was used to assess muscular strength. The apparatus consists of a box (21.5 by 22 by 23 cm) with a wire-mesh grid (10 by 10 cm) on top that can be inverted. Mice were placed on the wire mesh, which was then inverted, causing the animal to grip the wire. The latency to falling was recorded, with a 60-s cutoff time. For assessment of forelimb grip strength, a mouse was held by its tail and lifted so that its forepaws could grasp the wire grid of a grip-strength meter (O’Hara & Co.). It was then gently pulled backward by the tail until it released the grid. The peak force applied by the forelimbs was recorded. Each mouse was tested three times, and the highest value was used for statistical analysis.

### Hot-plate test

Sensitivity to a painful stimulus was assessed with the hot-plate test. Mice were placed on a hot plate maintained at 55.0° ± 0.2 °C and with a black anodized aluminum surface (Columbus Instruments, Columbus, OH). The latency to the first paw response (foot shake or paw lick) was recorded, with a cutoff time of 15 s.

### Rotarod test

Motor coordination and balance were evaluated with the rotarod test. Each mouse was placed on a rotating rod (Accelerating Rotarod; UGO Basile, Varese, Italy), and the latency to falling off the rod (the time that each animal was able to maintain its balance on the rod) during its acceleration from 5 to 40 rpm over 5 min was measured in three trials per day over two consecutive days. The mice were subjected to this test without any pretest training.

### Open-field test

Each mouse was placed in the corner of an open-field apparatus that consisted of a transparent Plexiglas chamber (40 by 40 by 30 cm) with a white plastic floor (Accuscan Instruments, Columbus, OH) and which was illuminated at 100 lx. The total distance traveled, vertical activity (rearing, measured by counting the number of photobeam interruptions), time spent in the central area (20 by 20 cm), and beam-break counts for stereotyped behavior were recorded over 120 min.

### Light–dark transition test

The apparatus for the light–dark transition test consisted of a cage with a white floor made of PVC (21 by 42 by 25 cm) that was divided into two sections of equal size by a partition with a door (O’Hara & Co.). The walls and roof of one chamber were made of white plastic and the chamber was brightly illuminated (390 lx), whereas the walls and roof of the other chamber were made of black plastic and it was maintained dark (2 lx). Mice were placed in the dark chamber and allowed to move freely between the two rooms with the door open for 10 min. The distance traveled in both chambers, the number of transitions between the two chambers, the time spent in each chamber, and the latency to entry into the light chamber were recorded with the use of ImageLD software.

### Elevated plus-maze test

The apparatus (O’Hara & Co.) consisted of two open arms (25 by 5 cm) and two enclosed arms of the same size with 15-cm-high transparent walls, and the arms were connected by a central square (5 by 5 cm). The arms and central square were made of white plastic plates and were elevated to a height of 55 cm above the floor. The likelihood of animals falling from the apparatus was minimized by the attachment of 3-mm-high plastic ledges to the open arms. Arms of the same type were arranged on opposite sides of the apparatus. Each mouse was placed in the central square of the maze facing one of the closed arms, and its behavior was recorded over 10 min. The total distance traveled, total number of arm entries, percentage of entries into the open arms, and percentage of time spent in the open arms were measured with the use of ImageEP software. Data for one mutant mouse that fell from the maze were excluded from analysis.

### Contextual and cued fear conditioning test

For assessment of fear-related learning and memory, a mouse was placed in and allowed to explore freely for 2 min a plastic chamber consisting of white lateral and transparent front, rear, and top surfaces (33 by 25 by 28 cm) and with a stainless-steel grid floor (bars 0.2 cm in diameter, spaced 0.5 cm apart) (O’Hara & Co.). A conditioned stimulus (CS) of 55-dB white noise was presented for 30 s and was followed by a mild foot shock (0.3 mA), which was administered during the last 2 s of the CS and served as the unconditioned stimulus (US). Two more CS-US pairings were presented with an interval of 2 min between each pair. A context test was conducted in the same chamber for 5 min both 1 and 28 days after conditioning. A cued test with an altered context was also performed on these days for 6 min in a triangular chamber (33 by 29 by 32 cm) that had walls and a floor made of white plastic and was located in a different sound-attenuating room. In the cued test, the mouse was allowed to move freely for 3 min and was then exposed to the auditory stimulus (55-dB white noise) for 3 min. The test chambers were equipped with a ceiling-mounted video camera connected to a computer for monitoring of mouse behavior. Images were captured at a rate of one frame per second. For each successive frame, the amount of area (pixels) within which the mouse moved was measured. When this area was below a certain threshold, the behavior was judged as “freezing.” When the area equaled or exceeded the threshold, the behavior was judged as “nonfreezing.” The optimal threshold (number of pixels) for judgment of freezing was determined by adjustment based on the degree of freezing measured by human observation. In each test, the percent freezing time and distance traveled were calculated automatically with the use of ImageFZ software. After each conditioning and context test, the plastic surface and grid floor of the chamber were wiped with hypochlorite solution and 65% ethanol, respectively. After each cued test, the walls and floor of the chamber were cleaned with hypochlorite solution.

### Acoustic startle response and prepulse inhibition test

A startle-reflex measurement system (O’Hara & Co.) was used to measure an acoustic startle response (ASR) elicited by a loud stimulus as well as prepulse inhibition of the startle response (PPI). Mice were placed in a PVC plastic cylinder. They were left undisturbed for 10 min and then subjected to test trials consisting of six trial types: two types of startle stimulus–only trial and four types of PPI trial. White noise of 110 or 120 dB (duration of 40 ms) was used as the startle stimulus for all trial types. A prepulse stimulus with an intensity of 74 or 78 dB (duration of 20 ms) was presented 100 ms before the onset of the startle stimulus. The four combinations of prepulse and startle stimuli were thus 74 and 110, 78 and 110, 74 and 120, and 78 and 120 dB. Mice were subjected to six blocks of the six trial types in a pseudorandom order such that each trial type was presented once within each block. The average intertrial interval was 15 s (range, 10–20 s). The startle response was recorded for 400 ms beginning with the onset of the startle stimulus. The peak startle amplitude was used as a dependent variable. The background noise level was 70 dB during all the test sessions. Percent PPI was calculated for each mouse according to the following formula: PPI (%) = 100 × {1 – [(ASR amplitude in prepulse + startle trial)/(ASR amplitude in startle stimulus–only trial)]}.

### Social interaction test in a novel environment

Two mice of the same genotype that had been housed in different cages were placed together in a box (40 by 40 by 30 cm) (O’Hara & Co.) and allowed to explore freely for 10 min. Analysis was performed automatically with the use of ImageSI software. Images were captured at a rate of three frames per second, and the distance traveled between two successive frames was determined for each mouse. If the two mice contacted each other and the distance traveled by them was > 10 cm, then the behavior was considered an active contact. The total duration of contacts, total number of contacts, total duration of active contacts, mean duration per contact, and total distance traveled were measured.

### Social interaction test in the home cage

The home cage–monitoring system consisted of a home cage (25 by 15 by 23.5 cm, interior dimensions) and a cage top equipped with an infrared video camera (O’Hara & Co.). Two mice of the same genotype that had been housed separately were placed together in the home cage. Images from the cage were captured at a rate of one frame per second. Social interaction was measured by counting the number of particles detected in each frame (two particles indicated that the mice were not in contact with each other, and one particle indicated contact between the two mice). The activity level of the mice was also measured by quantifying the number of pixels that changed between each pair of successive frames. The mean number of particles and mean activity level were calculated over 1-h intervals for 7 days, with the values for the first 3 days and the last 3 days being averaged for analysis of social behavior in a familiar environment. Analysis was performed automatically with the use of ImageHC software.

### Three-chamber tests of sociability and social novelty preference

The testing apparatus consisted of a rectangular, three-chambered box with a lid fitted with a video camera (O’Hara & Co.). The dividing walls of the box were made of transparent plastic, with small square openings (5 by 3 cm) allowing access to each chamber (20 by 40 by 47 cm). A small round wire cage (9 cm in diameter and 11 cm in height, with vertical bars 0.5 cm apart) was located in a corner of the left and right chambers. The test mice were first placed in the middle chamber and allowed to explore the entire apparatus for 10 min. It was then transferred to a clean holding cage, while a male C57BL/6 J mouse (stranger 1) that had had no prior contact with the test mouse was enclosed in one of the wire cages. The location of stranger 1 in the left versus right side chamber was systematically alternated between trials. The test mouse was then returned to the middle chamber of the test box and allowed to explore for 10 min (sociability test). After the test session, the test mouse was again placed in the holding cage, and a second unfamiliar male C57BL/6 J mouse (stranger 2) was enclosed in the remaining empty wire cage. The test mouse was returned to the middle chamber of the test box and now had a choice to explore either the already-investigated unfamiliar mouse or the novel unfamiliar mouse for 10 min (social novelty preference test). The time spent in each chamber and the time spent around each cage were automatically measured from images with the use of ImageCSI software.

### Porsolt forced swim test

A Plexiglas cylinder (20 by 10 cm) (O’Hara & Co.) filled with hypochlorite solution at 21° to 23 °C up to a height of 7.5 cm was placed in a white plastic chamber (44 by 32 by 49 cm, inside dimensions) (O’Hara & Co.). Each mouse was placed in the cylinder, and its immobility time was recorded over a 10-min test period. Images were captured at a rate of two frames per second with a video camera. For each pair of successive frames, the area (number of pixels) within which the mouse moved was measured. When this area was below a certain threshold, the mouse was judged to be immobile. When the area equaled or exceeded the threshold, the mouse was considered to be moving. The optimal threshold was determined by adjustment based on the degree of immobility measured by human observation. Immobility lasting < 2 s was not included in the analysis. Data acquisition and analysis were performed automatically with the use of ImageTS/PS software.

### Barnes maze test

The Barnes maze test was performed on “dry land,” a white circular surface with a diameter of 100 cm and with 12 holes equally spaced around the perimeter (O’Hara & Co.). The circular open field was elevated 75 cm from the floor. The apparatus was illuminated by fluorescent lights mounted on the ceiling of a sound-attenuating room, with an illumination level of ~ 850 lx in the center of the field. A variety of fixed extramaze clues surrounded the apparatus. A black Plexiglas escape box (17 by 13 by 7 cm) was located under one of the holes, designated the target hole, which is analogous to the hidden platform in the Morris water-maze task. The location of the target was consistent for a given mouse but was randomized across mice. In a training session, the mouse was placed in the center of the field and allowed to explore the maze freely. After it had entered the target hole, the mouse was left undisturbed in the escape box for 30 s. The training session was conducted in two trials each day for nine consecutive days. The maze was rotated daily, with the spatial location of the target remaining unchanged with respect to the visual room cues in order to prevent bias based on olfactory or proximal cues within the maze. The latency to reach the target hole, number of errors before reaching the target hole, distance traveled to reach the target hole, and number of omission errors (defined by a visit to the target hole without subsequent entry into the escape box) were automatically recorded with the use of ImageBM software. One and 30 days after the last training session, probe trials were conducted without the escape box for 180 s in order to assess spatial reference memory. In the probe trials, the time spent around each hole was measured.

### T-maze test

The spontaneous alternation task was conducted with an automatic T-maze apparatus (O’Hara & Co.) constructed of white plastic runways with 25-cm-high walls. The maze is partitioned into six areas by sliding doors that can be opened downward: stem of the T, straight runway, left and right arms, and connecting passageways from the arms to the stem of the T. Each mouse was subjected to sessions consisting of 10 trials per day (cutoff time, 50 min) for 3 days. Each trial consisted of a forced choice followed by a free choice. In the forced-choice run, the mouse was forced to enter either the left or right arm of the T-maze and was kept in the arm for 10 s. A free-choice run in which the mouse was allowed to choose one of the arms was then performed after a delay of 3, 10, 30, or 60 s. Choice by the mouse of the arm opposite that selected during the forced-choice run was considered a correct response, and the percentage of correct responses was calculated automatically with the use of ImageTM software.

### Data analysis

The applications for analysis of behavioral data (ImageLD, ImageEP, and ImageFZ, ImageSI, ImageHC, ImageCSI, ImageTS/PS, ImageTM) were based on ImageJ (http://rsb.info.nih.gov/ij) and developed by T. Miyakawa[41].

### Statistical analysis

Statistical analysis was performed with the use of BellCurve for Excel software (Social Survey Research Information, Tokyo, Japan). Normality of data was first assessed with the Shapiro–Wilk test, and homogeneity of variance between genotypes was examined with the F-test for each behavioral measure. If the normality assumption was not met, the Mann–Whitney U test was applied for comparisons between genotypes. If data were normally distributed and variance was homogeneous between genotypes, comparisons were performed with Student’s *t* test. If homogeneity of variance was not assumed, Welch’s *t* test was applied instead of Student’s *t* test. Two-way repeated-measures analysis of variance (ANOVA) was also conducted. All statistical analysis values, including ANOVA results, are included in Additional file 2: Table S2.

## Supplementary Information

**Additional file 1: Fig. S1.** Genomic sequences of PDZD8-KO mice. **a** Partial nucleotide sequence of the mouse *Pdzd8* ORF from exon 1 to exon 4. The sequences of exons 1, 2, 3, and 4 are shown in red, green, blue, and purple, respectively. The sequence corresponding to the SMP domain is highlighted in yellow. **b** ORF sequence of the Ex3d (*Pdzd8*^*tm1b*^) mutant (from EUCOMM) [25]. The sequences of exons 1, 2, and 4 are shown in red, green, and purple, respectively. The sequence corresponding to the SMP domain is highlighted in yellow, and the stop codon is indicated with a double underline. The predicted protein contains the first methionine, TM domain, and SMP domain.

**Additional file 2: **.

## Data Availability

The datasets used and/or analyzed during the current study are available from the corresponding author on reasonable request.

## Abbreviations

PDZD8: PDZ domain containing 8
CE: Cholesteryl ester
ER: Endoplasmic reticulum
SMP: Synaptotagmin-like mitochondrial lipid binding protein
ID: Intellectual disability
PTSD: Posttraumatic stress disorder
TM: Transmembrane
KO: Knockout
sgRNA: Single guide RNA
ORF: Open reading frame
WT: Wild-type
