# Supplementary material for: PDZD8-deficient mice manifest behavioral abnormalities related to emotion, cognition, and adaptation due to dyslipidemia in the brain
Source: Mol Brain. 2023 Jan 19;16:11. doi: 10.1186/s13041-023-01002-4 (PMC9854033; doi:10.1186/s13041-023-01002-4)
Supplement: Supplementary file 1 — Additional file 1: Fig. S1. Genomic sequences of PDZD8-KO mice. a Partial nucleotide sequence of the mouse Pdzd8 ORF from exon 1 to exon 4. The sequences of exons 1, 2, 3, and 4 are shown in red, green, blue, and purple, respectively. The sequence corresponding to the SMP domain is highlighted in yellow. b ORF sequence of the Ex3d (Pdzd8tm1b) mutant (from EUCOMM) [25]. The sequences of exons 1, 2, and 4 are shown in red, green, and purple, respectively. The sequence corresponding to the SMP domain is highlighted in yellow, and the stop codon is indicated with a double underline. The predicted protein contains the first methionine, TM domain, and SMP domain. [file 13041_2023_1002_MOESM1_ESM.pdf]

a

Mouse *Pdzd8* ORF (1-1134 b):

ATG GGG CTG CTG CTC CTG ATC CTG GCG TCG GCC GTG CTG GGC TCG TTC CTC
ACG CTG CTC GCA CAG TTC CTG CTA CTC TAC CGC AGA CAG CCC GAG CCG CGG
GCG GAC GAA GCG GCC CGC GCG GGC GAC GGC TTC CGC TAC CTT AAG CCG GTG
CCG GGC CTG CCC CTC AGG GAG TAC CTT TAT GGC GGC GGC GCC GAG GAG CTC
GCT GCT TGC TCC TCC GAG GCC GGC GCC AGC TCG ACC CCG ACC CCC GAC AGC
CCG GCC CCG CCG ACG CTG GAG ACC TGC TAC TTC CTC AAC GCC ACC ATC CTG
TTC CTG TTC CGG GAG CTG CGG GAC ACC GCG CTC GCC CGC CGC TGG GTC ACC
AAG AAG ATC AAG GTG GAG TTC GAG GAG CTG CTG CAG ACC AAG ACG GCC GGC
CGC CTG CTG GAG GGG CTG AGT CTG CGC GAC GTG TTC CTG GGC GAC ACG GTG
CCC TTC ATC AAG ACC ATC CGG CTG GTG CGG CCC GTG GTG GCC TCG GGC ACC
GGC GAG CCC GAC GAC CCC GAC GGG GAC GCG CTG CCC GCC ACC TGC CCG
GAG GAG CTG GCC TTT GAA GCG GAG GTG GAG TAC AAC GGC GGT TTC CAC CTG
GCC ATC GAC GTG GAC CTG GTG TTT GGC AAG TCC GCC TAC CTG TTC GTG AAG
CTG TCT CGC GTG GTG GGG AGG CTG CGC TTC GTC CTC ACC CGC GTG CCC TTC
ACC CAC TGG TTC TTC TCC TTC GTG GAG GAC CCG CTG ATT GAC TTC GAG GTG
CGC TCC CAG TTC GAG GGG CGG CCC ATG CCC CAG CTC ACC TCC ATC ATC GTC
AAC CAG CTC AAG AAG ATC ATC AAG CGC AAG CAC ACC CTG CCC AGT TAC AAG
ATC AGG TTT AAG CCG TTT TTT CCA TAC CAA GCC TTG CAA GGA TTT GAA GAA GAT
GAA GAA CTT ATC CAT ATT CAA CAA TGG GCA CTT ACT GAA GGC CGG CTT AAA GTT
ACA TTG TTA GAA TGT AGC AGG TTA TTC ATT TTT GGA TCC TAT GAC AGA GAA ACA
AAT GTT CAT TGC ACA CTT GAG CTG AGC AGT GGT GTT TGG GAA GAA AAA CAA AGG
AGT TCT ATT AAG ACG GTT GAA TTA ATA AAA GGG AAT TTA CAA AGT GTC GGA CTT

Ex1
Ex2
Ex3
Ex4
SMP domain

b

Ex3d ORF:

ATG GGG CTG CTG CTC CTG ATC CTG GCG TCG GCC GTG CTG GGC TCG TTC CTC
ACG CTG CTC GCA CAG TTC CTG CTA CTC TAC CGC AGA CAG CCC GAG CCG CGG
GCG GAC GAA GCG GCC CGC GCG GGC GAC GGC TTC CGC TAC CTT AAG CCG GTG
CCG GGC CTG CCC CTC AGG GAG TAC CTT TAT GGC GGC GGC GCC GAG GAG CTC
GCT GCT TGC TCC TCC GAG GCC GGC GCC AGC TCG ACC CCG ACC CCC GAC AGC
CCG GCC CCG CCG ACG CTG GAG ACC TGC TAC TTC CTC AAC GCC ACC ATC CTG
TTC CTG TTC CGG GAG CTG CGG GAC ACC GCG CTC GCC CGC CGC TGG GTC ACC
AAG AAG ATC AAG GTG GAG TTC GAG GAG CTG CTG CAG ACC AAG ACG GCC GGC
CGC CTG CTG GAG GGG CTG AGT CTG CGC GAC GTG TTC CTG GGC GAC ACG GTG
CCC TTC ATC AAG ACC ATC CGG CTG GTG CGG CCC GTG GTG GCC TCG GGC ACC
GGC GAG CCC GAC GAC CCC GAC GGG GAC GCG CTG CCC GCC ACC TGC CCG
GAG GAG CTG GCC TTT GAA GCG GAG GTG GAG TAC AAC GGC GGT TTC CAC CTG
GCC ATC GAC GTG GAC CTG GTG TTT GGC AAG TCC GCC TAC CTG TTC GTG AAG
CTG TCT CGC GTG GTG GGG AGG CTG CGC TTC GTC CTC ACC CGC GTG CCC TTC
ACC CAC TGG TTC TTC TCC TTC GTG GAG GAC CCG CTG ATT GAC TTC GAG GTG
CGC TCC CAG TTC GAG GGG CGG CCC ATG CCC CAG CTC ACC TCC ATC ATC GTC
AAC CAG CTC AAG AAG ATC ATC AAG CGC AAG CAC ACC CTG CCC AGT TAC AAG
ATC AGG TTT AAG CCG TTT TTT CCA TAC CAA GCC TTG CAA GGA TTT GAA GAA GAT
GAA GAA CTT ATC CAT ATT CAA CAA TGG GCA CTT ACT GAA GGC CGG CTT AAA GTT
ACA TTG TTA GAA TGT AGC AGT TGA

Ex1
Ex2
Ex4
SMP domain

Kurihara et al, Fig. S1
